# Isolated Case of Marburg Virus Disease, Kampala, Uganda, 2014

**DOI:** 10.3201/eid2306.170047

**Published:** 2017-06

**Authors:** Luke Nyakarahuka, Joseph Ojwang, Alex Tumusiime, Stephen Balinandi, Shannon Whitmer, Simon Kyazze, Sam Kasozi, Milton Wetaka, Issa Makumbi, Melissa Dahlke, Jeff Borchert, Julius Lutwama, Ute Ströher, Pierre E. Rollin, Stuart T. Nichol, Trevor R. Shoemaker

**Affiliations:** Uganda Virus Research Institute, Entebbe, Uganda (L. Nyakarahuka, J. Lutwama);; US Centers for Disease Control and Prevention, Entebbe (J. Ojwang, A. Tumusiime, S. Balinandi, J. Borchert, T.R. Shoemaker);; Centers for Disease Control and Prevention, Atlanta, Georgia, USA (S. Whitmer, U. Ströher, P.E. Rollin, S.T. Nichol);; Public Health Emergency Operations Center, Kampala, Uganda (S. Kyazze, S. Kasozi, M. Wetaka, I. Makumbi, M. Dahlke)

**Keywords:** viruses, Marburg virus, Kampala, Uganda, viral hemorrhagic fever, fruit bat, Rousettus aegyptiacus, zoonoses

## Abstract

In September 2014, a single fatal case of Marburg virus was identified in a healthcare worker in Kampala, Uganda. The source of infection was not identified, and no secondary cases were identified. We describe the rapid identification, laboratory diagnosis, and case investigation of the third Marburg virus outbreak in Uganda.

Marburg virus disease (MVD) is caused by Marburg virus (MARV; family *Filoviridae*, which also includes Ebola viruses). The disease was first discovered in 1967 in Marburg and Frankfurt, Germany, after laboratory workers were infected from monkeys imported from Uganda (*1*). Thereafter, sporadic cases and outbreaks of MVD have been documented in South Africa, Kenya, the Democratic Republic of the Congo, Angola, Uganda, the Netherlands, and the United States (*2*). MVD remains of great public health importance because of the case-fatality rate, which can be as high as 90%, and documented human-to-human transmission, with associated socioeconomic consequences.

Uganda has experienced previous outbreaks of MVD, resulting in fatalities and socioeconomic effects from loss of tourism. The first recorded outbreak in Uganda occurred in the Kamwenge district in 2007, where 4 MVD cases were confirmed in miners at the Kitaka mine (*3*). A second, larger outbreak occurred in the western Uganda districts of Kabale, Ibanda, and Kamwenge in 2012 (*4*) and was also linked to mining activity in the Ibanda district. In addition, tourists from the United States and the Netherlands were infected with MARV in western Uganda when they visited Python Cave in Queen Elizabeth National Park in 2008 (*5*,*6*). Both Python Cave and the Kitaka mine are inhabited by Egyptian fruit bats (*Rousettus aegyptiacus*), the host reservoir of MARV (*7*).

In 2014, a fatal case of MVD occurred in Uganda. We report here on the field and laboratory investigation of this case, including possible sources of infection.

## The Study

**Figure 1:**
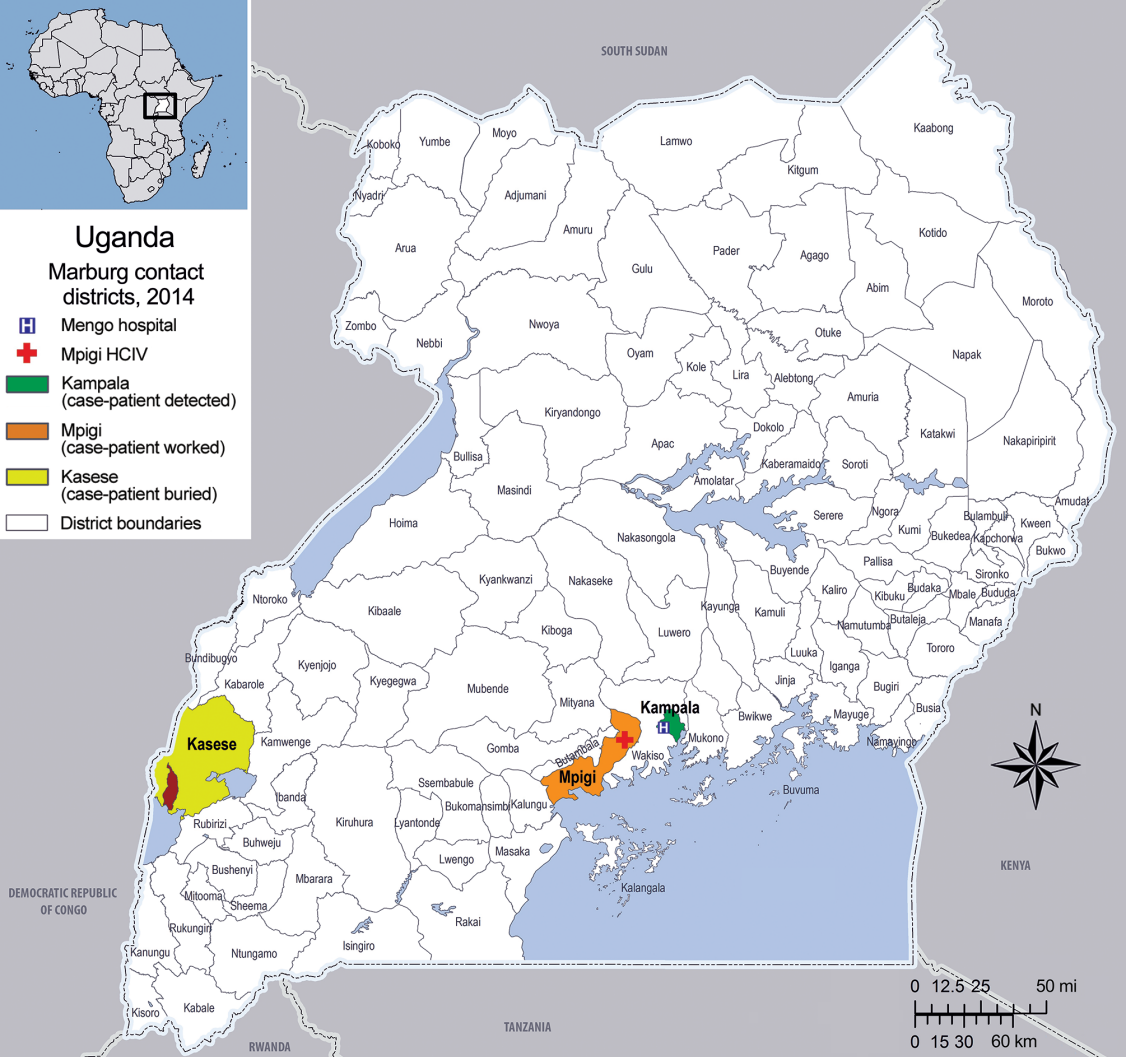
Locations where patient with confirmed Marburg virus disease lived, worked, and was buried, Kampala, Uganda, 2014. Inset map shows location of Uganda in Africa.

On September 23, 2014, a healthcare worker was admitted to Mengo Hospital in Kampala, Uganda, with a febrile illness suspected to be viral hemorrhagic fever (VHF). The patient, a 30-year-old man, was a radiographer who worked at Mengo Hospital and part-time at Mpigi Health Center IV in the Mpigi district, ≈30 km south of Kampala (Figure 1). His symptoms began on September 17, 2014. A rapid diagnostic test result was positive for malaria, and the patient was given intravenous ceftriaxone, 5% dextrose, and artesunate. However, his condition continued to deteriorate. 

On September 26, he began to display hemorrhagic signs, notably profuse bleeding from body orifices. Clinical findings included fever (temperature 38.4°C), nausea, vomiting, diarrhea, musculoskeletal pain, abdominal pain, headache, sore throat, difficulty swallowing, difficulty breathing, anorexia, bleeding from the nose, bloody stools, vomiting blood, and upper gastrointestinal tract bleeding. He died on September 28.

Whole blood and serum samples collected on September 28 were sent to the Uganda Virus Research Institute (UVRI)/US Centers for Disease Control and Prevention (CDC) VHF laboratory in Entebbe for testing on September 30. We performed diagnostic testing by real-time reverse transcription PCR (RT-PCR) targeting the MARV NP gene, antigen detection, and MARV IgM ELISA on the suspected sample as described (*8*). We detected MARV by RT-PCR, but antigen detection and IgM serologic results were negative.

We obtained independent confirmation from duplicate whole blood and serum samples by using RT-PCR primers and probes targeting MARV VP40 gene and a commercial filovirus PCR screening assay (Altona Diagnostics, Hamburg, Germany) (*9*) (Table). On the basis of the positive RT-PCR results, we confirmed MVD in this patient. We shipped specimens to the CDC Viral Special Pathogens Branch, Division of High-Consequence Pathogens and Pathology, National Center for Emerging and Zoonotic Infectious Diseases (Atlanta, GA, USA), for further testing, including virus isolation and sequencing. A virus isolate (812601) was generated from the clinical specimen after a single passage in cell culture (Vero E6).

**Table:** Primers used to generate Marburg virus-specific cDNA fragments for whole-genome sequencing of isolates from Uganda

Genome fragment	Primer set	Primer sequence, 5’→3’	Region amplified	Region size, kbp
1	63F	TGA CAT TGA GAC TTG TCA GTC	64-4998	4.9
	MARB-4997R	GCT TGA TTT CCT TCA CGC		
2	3005F	AAG TCA GCG AGG GGT TGA TGA CTG GAA AAG	2970-6438	3.5
	6426R	TGC TAT GTT CCC TTC AGT GAA GAC		
3	6101F	AGA AAA CAG AAG ACG TCC ATC TGA TG	6065-9405	3.3
	9405R	ACT TAA TGC TGC ACG AAG TGA TG		
4	7567F	TGG CCC TGG AAT IGA AGG ACT C	7548-10571	3.0
	10571R	AGC ATA TGA ACA ATA GAT C		
5	36F	GTA CCT CTA AGG AAA ATC ATG AAG	9979-15442	5.4
	57R	GTT GAT ATA ATT GCA CGT GTA GAT		
6	12006F	TTG CCA GAA GGA TAA AAG GAC AAA GAG	11953-15486	3.5
	15516R	ATT TTG GAA GAT TAT ATT ACT ATC		
7	15519F	TGG ACG ATA GGA AAT CGA GCA C	15108-19111	4.0
	19155R	TGG ACA CAC TAA AAA GAT G		

After laboratory confirmation, a multidisciplinary team from UVRI and CDC-Uganda performed the initial outbreak investigations at Mengo Hospital and Mpigi Health Center IV. The investigation team provided the outbreak case definition, and details of case contacts were obtained from both health facilities. An investigation was also performed in Kasese District, where the health worker was taken for burial. In addition, an ecologic investigation was conducted to identify roosting sites nearby for the potential presence of *R. aegyptiacus* bats.

We created an outbreak database by using the Epi Info Viral Hemorrhagic Fever outbreak management application (*10*). All case and contact data were managed by the Uganda Ministry of Health Public Health Emergency Operations Center. We identified 197 close contacts, who were followed for 21 days. During the course of follow-up, 33 (16.2%) of the 197 contacts converted to suspected case-patients by exhibiting clinically compatible signs or symptoms matching the outbreak case definition. Blood samples from suspected case-patients were tested at the UVRI/CDC laboratory in Entebbe; all were negative for MARV by RT-PCR and serologic analysis.

## Conclusions

We describe the second single-case filovirus outbreak detected in Uganda; a case of infection with *Sudan ebolavirus* was reported in Luwero District in 2011 (*11*). The investigation was unable to identify a conclusive source of infection, including evidence of the natural reservoir host near where the infected patient was working or residing or potential cases in persons who visited health centers before this case was confirmed. No secondary cases arose from contact with the initial case-patient. 

The patient tested positive for malaria but later tested positive for MARV by RT-PCR and serologic analysis. These findings indicate that co-infection of viral hemorrhagic fever (VHF) and other tropical infectious diseases can confound diagnosis and delay early detection, potentially resulting in large outbreaks that are much harder to control, as was seen during the 2014–2015 Ebola virus outbreak in West Africa (*12*). This finding emphasizes the need for continued surveillance and awareness even when other, more common pathogens are initially suspected.

The finding of no secondary cases in this investigation can be attributed, in part, to use of infection control practices and personal protective equipment when first encountering a suspected VHF case. Because Uganda has experienced 10 VHF outbreaks since 2011, increased awareness and use of personal protective equipment and infection control practices have greatly limited secondary transmission, especially in the healthcare setting. Routine use of gloves, protective gowns, and chlorine is now more common in lower-level healthcare facilities in Uganda; these protective products were used at both Mengo Hospital and Mpigi Health Center IV.

**Figure 2:**
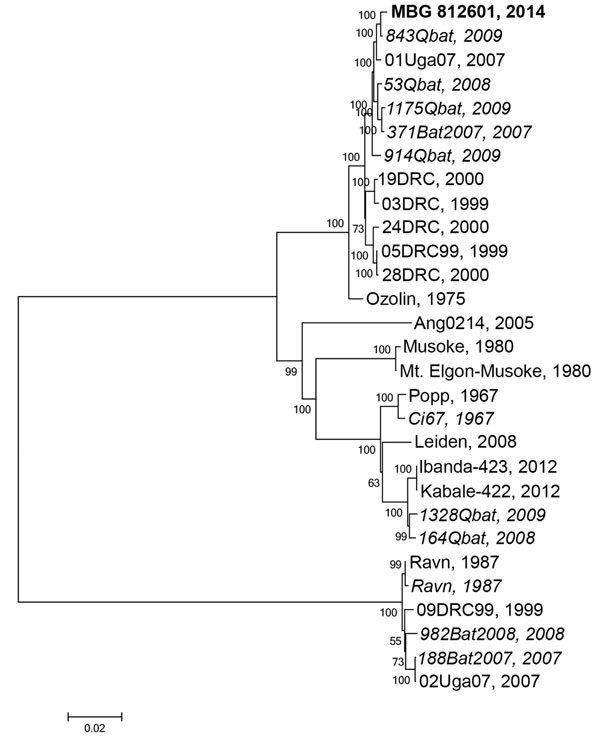
Phylogenetic tree comparing complete or nearly complete Marburg virus (MARV) genomes sequenced from bat and human sources in Uganda. A consensus whole-genome sequence was assembled by mapping reads to the reference MARV sequence NC_001608 using CLC Genomics Workbench (Waltham, MA, USA). A phylogenetic tree was constructed using MEGA6.06 (http://www.megasoftware.net). Viral sequences acquired from human sources are in standard type, and viral sequences acquired from bats are italicized; the sequence from the human case-patient described in this study, MBG 812601 2014, is in bold. Evolutionary history was inferred using the maximum-likelihood method based on the Tamura-Nei model with MEGA6.06. The tree is drawn to scale, with branch lengths measured in the number of substitutions per site. Values at nodes represent bootstrap values following 1,000 replicates. Scale bar represents substitutions per site. GenBank accession numbers used in this tree are KP985768, JX458855.1, FJ750957.1, JX458852.1, JX458854.1, FJ750958.1, JX458856.1, JX458828.1, JX458826.1, JX458834.1, DQ447651.1, JX458846.1, AY358025.2, DQ447657.1, Z12132.1, NC_001608.3, Z29337.1, EF446132.1, JN408064.1, KC545388.1, KC545387.1, DQ447649.1, EF446131.1, DQ447652.1, FJ750956.1, FJ750955.1, and FJ750953.1.

The full genomic sequence of this MARV isolate (812601; GenBank accession no. KP985768) falls into a cluster that consists mostly of MARV sequences isolated from bats. The closest related sequence was obtained from MARV isolated from a juvenile male *R. aegyptiacus* bat (Q843) captured in August 2009 in Python Cave, Queen Elizabeth National Park (Figure 2). Other viral sequences from bat specimens in this clade were from bats collected from either Python Cave or the Kitaka mine during 2007–2009. The most closely related human sequence (01Uga07) was from a miner who worked in the Kitaka mine in July 2007 (*3*,*7*). Because of the wide genetic diversity of MARVs, there is no definitive way to identify where this patient may have become infected.

We do not know why the patient did not transmit MARV to any of his close contacts. We can assume that none of the contacts had substantial exposure to the patient while he was infectious. The relatively small size of this and previous filovirus outbreaks in Uganda can be attributed to enhanced VHF surveillance, rapid case identification, laboratory testing, and investments from the global health security agenda in rapid sample transportation to the national VHF reference laboratory for diagnostic testing (*13*). VHF surveillance continues to be a top priority for Uganda, and the VHF surveillance program continues to play a crucial role in detecting, responding to, and helping to control these outbreaks.
